# The mechanism of triptolide in the treatment of connective tissue disease-related interstitial lung disease based on network pharmacology and molecular docking

**DOI:** 10.1080/07853890.2022.2034931

**Published:** 2022-02-08

**Authors:** Wen Zhu, Yehui Li, Junjie Zhao, Yifan Wang, Yixi Li, Yue Wang

**Affiliations:** a Department of Rheumatology, Nanjing University of Chinese Medicine, Nanjing, China; b Department of Pneumology, Affiliated Hospital of Nanjing University of Chinese Medicine, Nanjing, China; c Jiangsu Province Hospital of Chinese Medicine, Nanjing, China

**Keywords:** Tripterygium wilfordii, Triptolide, CTD-ILD, Network pharmacology, Molecular docking

## Abstract

**Background:**

Interstitial lung disease (ILD) is associated with substantial morbidity and mortality, which is one of the key systematic manifestations of connective tissue disease (CTD). Tripterygium wilfordii, known as Leigongteng in Chinese, has been applied to treat connective tissue disease-related interstitial lung disease (CTD-ILD) for many years. Triptolide is a key effective component from Tripterygium wilfordii. But the molecular mechanism of Triptolide for treating CTD-ILD is not yet clear.

**Methods:**

Gaining insight into the molecular mechanism of Triptolide intervention CTD-ILD, we used the method of network pharmacology. And then we conducted drug-target networks to analyse the potential protein targets between Triptolide and CTD-ILD. Finally, AutoDock Vina was selected for molecular docking.

**Results:**

By analysing the interaction genes between Triptolide and CTD-ILD, 242 genes were obtained. The top 10 targets of the highest enrichment scores were STAT3, AKT1, MAPK1, IL6, TP53, MAPK3, RELA, TNF, JUN, JAK2. GO and KEGG enrichment analysis exhibited that multiple signalling pathways were involved. PI3K-Akt, multiple virus infections, cancer signalling, chemokine, and apoptosis signalling pathway are the main pathways for Triptolide intervention CTD-ILD. And it is related to various biological processes such as inflammation, infection, cell apoptosis, and cancer. Molecular docking shows Triptolide can bind with its target protein in a good bond by intermolecular force.

**Conclusions:**

This study preliminarily reveals the internal molecular mechanism of Triptolide interfere with CTD-ILD through multiple targets, multiple access, validated through molecular docking.

**KEY MESSAGES**
- Triptolide intervention CTD-ILD, which are related to various biological processes such as inflammation, infection, cell apoptosis, and cancer.
- PI3K-Akt, multiple virus infections, and apoptosis signalling pathway are the main pathways for Triptolide intervention CTD-ILD.
- Triptolide can bind with related target protein in a good bond by Intermolecular force, exhibiting a good docking activity.

## 1. Introduction

Interstitial lung disease (ILD) [1], associated with significant morbidity and mortality, is a common manifestation and amongst the leading causes of morbidity and mortality in patients with connective tissue disease (CTD) [2]. CTD-ILD, which is characterized by immune-mediated tissue injury that can involve the lungs, has a mix of inflammatory and fibrosis [2]. ILD along with multicompartment lung involvement including airways, pleural and pulmonary vascular disease, may contribute to the aetiology of their respiratory impairment and potential responses to therapy [3]. Both the innate and adaptive immune systems can induce the development of fibrosis [4]. Following the injury, wound-healing responses are accompanied. If sustained and deregulated, pathological fibrogenesis then occurs, whereby the rate of new collagen synthesis exceeds the rate of collagen degradation, culminating in the accumulation of collagen over time [5]. During the development and evolution of this disease, and immune imbalance is the main pathological factor, and a variety of immune cells, such as T cells, B cells, macrophages, etc. are involved in the occurrence and development of this disease [6,7]. Controlling a variety of pro-inflammatory factors and restoring immune homeostasis is the main direction of inhibiting the progression of this disease from inflammation to fibrosis [8]. Among them, FGF, VEGF, TGF, PDGF were thought the critical pro-inflammatory factors [8]. But there is currently little high-level evidence to guide the management of CTD-ILD. Novel approaches involving biological agents, antifibrotic drugs, and even stem cell transplants have been introduced for CTD-ILD treatment, although specific pulmonary benefit has not been conclusive. Thus, further efforts are still urgently needed to develop novel strategies to prevent this refractory respiratory disease.

Tripterygium wilfordii is a clinically common Chinese herbal medicine, which has been used to treat clinical diseases for more than 2000 years in China. Because of its good immunosuppressive effect in the fields of metabolic diseases [9], kidney diseases [10], rheumatic diseases [11], inflammatory bowel diseases [12], and so on [13], *Tripterygium wilfordii* hance has been paid much attention by scholars at home and abroad. Triptolide is a major active natural product isolated from the medicinal plant *Tripterygium wilfordii*, which has exhibited to have good anti-inflammatory, immunosuppressive and anti-fibrosis therapeutic effects [9], but its molecular mechanism of action is still unknown. Network pharmacology is an emerging discipline based on the network of disease-gene-drug targets [14], based on network interaction to study the basic biological knowledge of TCM can provide a deep insight or scientific evidence for the discovery of TCM, and help us to clarify the pharmacological mechanism of active ingredients of TCM at the level of biomolecule [15]. Network pharmacology is gradually becoming a holistic and efficient tool to describe the complex interactions between drugs and biological systems including the human organs, diseases, metabolic pathways, and target proteins from a network perspective [16].

**Figure 1.**
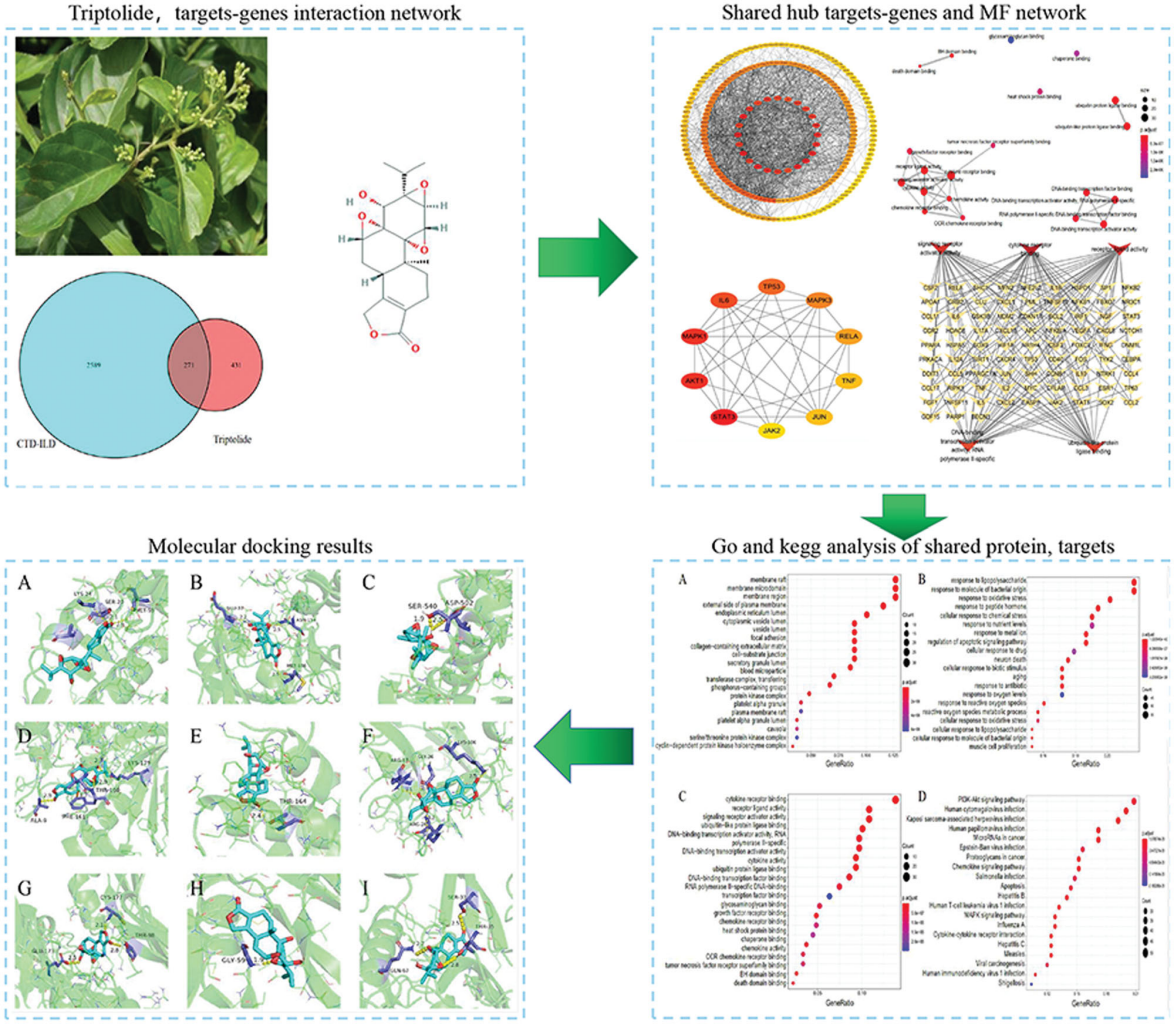
Workflow of the network pharmacology to identify Triptolide targets in CTD-ILD.

In this study, we constructed a network pharmacological model of Triptolide and systematically analysed the potential anti-CTD-ILD mechanism of Triptolide. Our studies revealed the potential mechanisms which may contribute to the occur of CTD-ILD. To confirm the predicted result, we provide a molecular docking model with potential targets and known targets. The detailed procedures can be seen in Figure 1.

## 2. Materials and methods

### 2.1. *Screening* of triptolide-related disease targets

All targets of Triptolide were gathered by using the following five database: Traditional Chinese Medicine Systems Pharmacology Database and Analysis Platform (http://lsp.nwu.edu.cn/index.php) [17], the Comparative Toxicogenomics Database (CTD) [18] (http://ctdbase.org/), GeneCards database (https://www.genecards.org) [19,20], STITCH (http://stitch.embl.de/) [21]and SymMap database (https://www.symmap.org/) [22]. Organism equal to Homo sapiens was limited. After removing the duplicated genes, related genes were returned.

### 2.2. Gene screening of CTD-ILD related targets

Using “Connective Tissue Disease-associated with Interstitial Lung Diseases” as the search keywords, we searched OMIM database (http://www.omim.org), Genebank database (https://www.ncbi.nlm.nih.gov/genbank), GeneCards database (https://www.genecards.org) to excavate potential targets associated with CTD-ILD. Access the DrugBank database (https://www.drugbank.ca) to find drug targets for approved intervention CTD-ILD [23]. The 4 disease database targets were combined and the repeat value was deleted to get the CTD-ILD related targets.

### 2.3. Construction of protein-protein interaction (PPI) network

Using R X 64 4.0.2 software, the intersection of the Triptolide target and related CTD-ILD target was obtained, then the Venn diagram was drawn. Intersection targets were extracted and submitted to STRING database (https://string-db.org) to build a protein-protein interaction (PPI) network [24]. The species type was set to “Homo sapiens”, the minimum interaction threshold was set to “highest confidence” (>0.9), and the rest were set as the default. Finally, the PPI result was imported into Cytoscape 3.8.0 software to construct a PPI network.

### 2.4. Hub gene analysis

Hub gene of PPI network of Triptolide against CTD-ILD was calculated by MCC algorithm in Cytohubba plugin of CytoScape [25], then the related protein targets network was constructed. Finally, the core targets of the top 10 were also exhibited.

### 2.5. Go and KEGG enrichment analysis

To clarify the role of target proteins interacting with Triptolide target genes in gene function and signalling pathways, we conducted GO and KEGG enrichment analysis of potential targets of Triptolide intervention CTD-ILD by R Software (R 4.0.2 for Windows). Save the data results and use R software for visual analysis.

### 2.6. Molecular docking

Molecular docking was performed among the top 5 potential target proteins and known 4 relative proteins of ILD with Triptolide. The 3D structure of the target protein was downloaded from the PDB database (https://www.rcsb.org). The water molecules and the original ligands were removed from the target protein through PyMOL, Later, the target proteins were imported into AutoDock Tools 1.5.6 for hydrogenation, charge calculation, and non-polar hydrogen combination, and then the result was stored in PDBQT format. Set the size of Grid Box to 40 × 40 × 40. Finally, run AutoDock Vina using CMD command characters for molecular docking [26], and use PyMOL to visualize the results.

## 3. Result

### 3.1. Acquisition of the main protein targets of triptolide

By searching the following databases: TCMSP, CTD, GeneCards, STITCH, and SymMap database, confining the result to “Homo sapiens”, 702 genes related with Triptolide were collected by using the term “Connective Tissue Disease-associated Interstitial Lung Diseases”.

### 3.2. Acquisition of CTD-ILD related targets

In Genecards database, the higher the Score value is, the closer the relationship is to the disease. When there are too many targets, the target with a score greater than the median is a CTD-ILD-related target. The maximum score of the target obtained by GeneCards is 166.33, the minimum score is 0.38, and the median is 17.64. Therefore, the genetic target with a Score of >17.64 is considered a CTD-ILD-related target. Then 1764 targets are obtained after screening. After searching the GenBank database, we found 86 related genes. A total of 1294 targets were obtained from the Genemap database. The DrugBank database search is complemented, and about 12 targets for Nidanib were obtained. Results were merged and the duplicate genes were eliminated, then 2860 CTD-ILD related targets were obtained.

### 3.3. Venn diagram and PPI network construction

**Figure 2.**
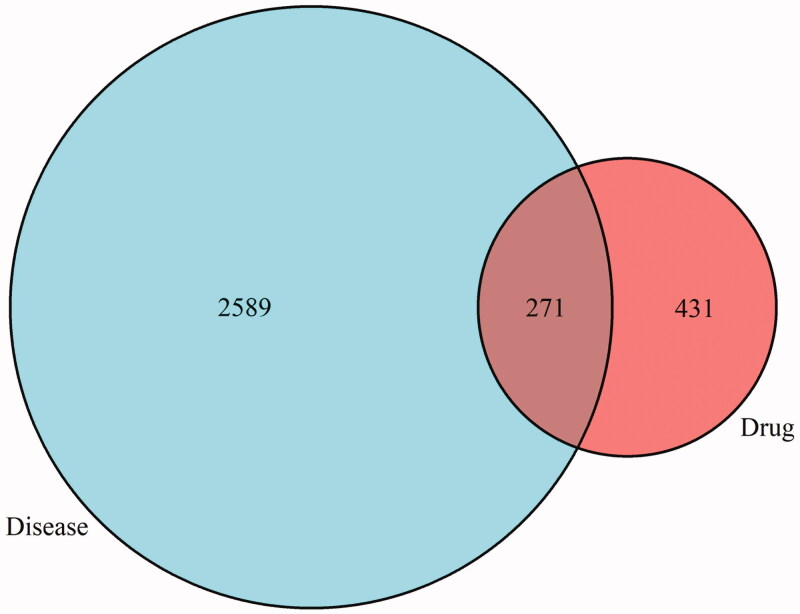
The Venn diagram of Triptolide and CTD-ILD targets.

**Figure 3.**
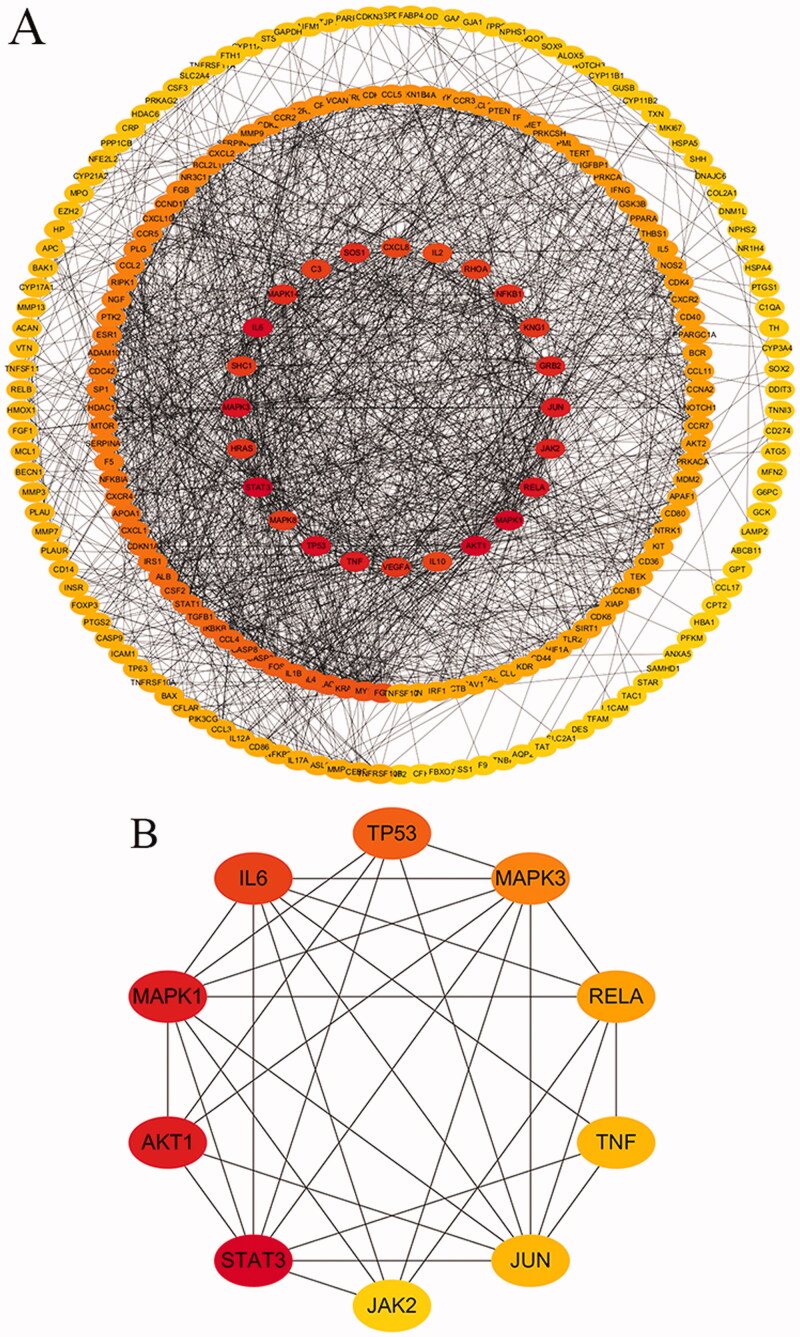
Against CTD-ILD targets network of Triptolide, the target genes are sorted according to a degree. (A) The degree of the outermost circle is 0–20, the middle circle is 21–40 , the smallest circle is 41–100. (B) Cytohubba, the plug-in of Cytoscape, was used to analyse the top 10 hub gene network of target proteins by MCC algorithm, in which gray value represents the importance in the network.

The intersection of Triptolide targets and CTD-ILD disease targets was taken, and Venn diagram was drawn by R software to obtain 271 intersection targets (Figure 2). The target proteins that act with their corresponding ingredients were submitted to STRING version 11.0 (http://string-db.org/) for PPI network construction, and high confidence of protein interaction data with a score >0.9 was selected. Removing free proteins that do not interact, There were 242 shared proteins between Triptolide and CTD-ILD. The protein-protein interactions networks suggested that 242 proteins and 1628 interactions (edges) could potentially interact in the intersection targets between Triptolide and CTD-ILD (Figure 3(A)).

### 3.4. Screening of hub gene and topological network analysis

Based on Cytohubba, the plug-in of Cytoscape, the Hub gene was screened in the interaction network. We used the MCC algorithm to find out the top 10 Hub genes of target proteins, and the Hub gene network diagram was constructed. Based on network topological analysis, the average betweenness centrality was 0.01 and the average degree of freedom was 13.45, signalling hub connectivity between genes. A total of 98 genes produced betweenness centrality and degree of freedom values above the mean, but only the top 10 genes are exhibited (Table 1). We can conclude that STAT3, AKT1, MAPK1, IL6, TP53, MAPK3, RELA, TNF, JUN, JAK2 are the important targets for Triptolide intervention CTD-ILD (Figure 3(B)).

**Table 1.** Target proteins with potentially critical roles in Triptolide treatment of CTD-ILD.

NO.	Gene abbre*via*tion	Betweenness centrality	Degree
1	STAT3	0.093621689	63
2	MAPK1	0.068443793	54
3	AKT1	0.065861966	54
4	IL6	0.080875215	52
5	TP53	0.078781834	51
6	MAPK3	0.034771135	50
7	RELA	0.042217185	46
8	JUN	0.035870028	42
9	TNF	0.031940891	42
10	JAK2	0.028176151	38

### 3.5. Go and KEGG analysis

**Figure 4.**
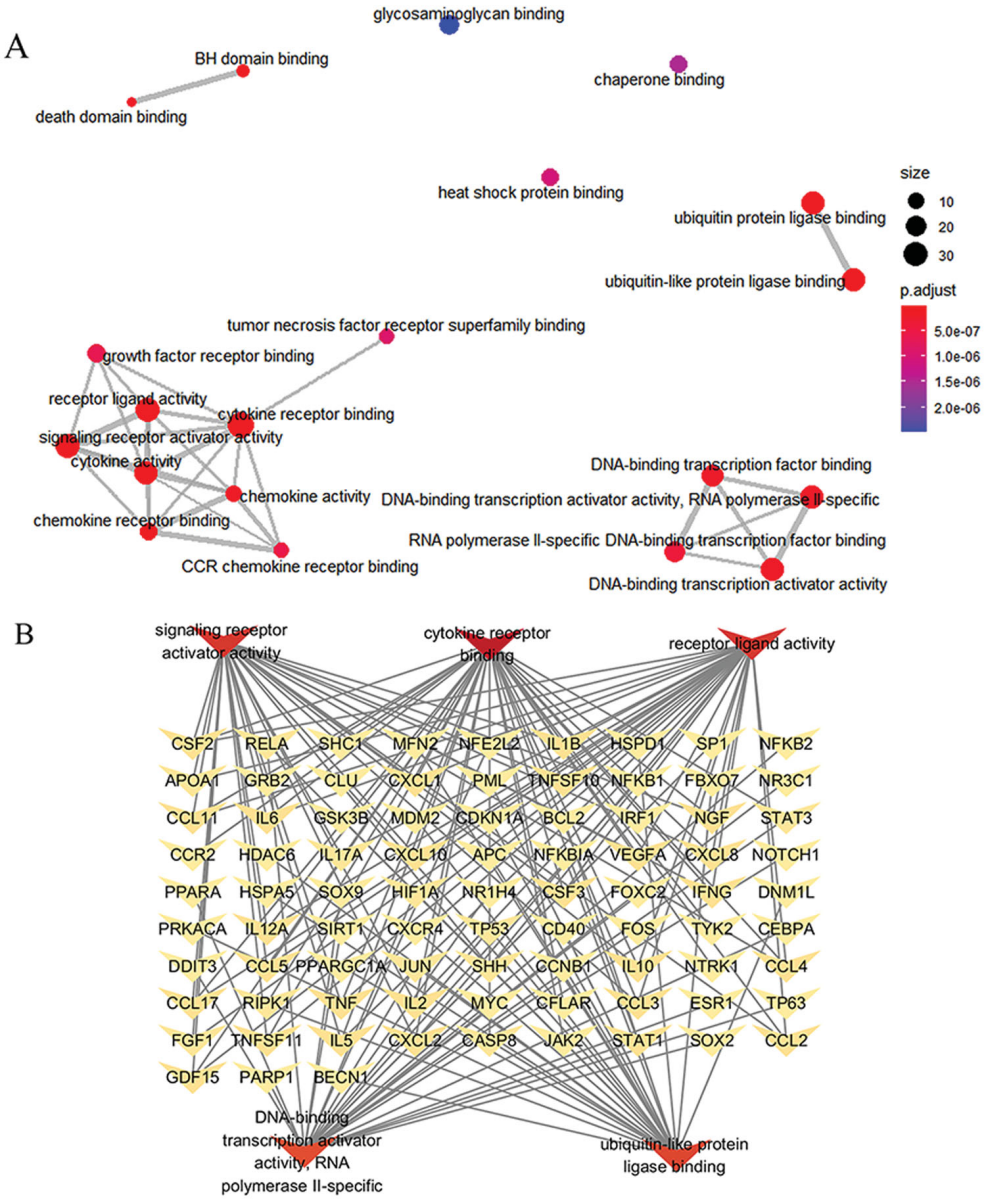
Topological network schematic of proteins targeted by Triptolide and associated with CTD-ILD. (A) Interaction networks between enriched molecular functions are analysed by enrichMap in the clusterProfiler package. The scales indicated different thresholds of adjusted *p*-values, and the sizes of the dots represent the gene count of each term. (B) Sub-network showing important genes in the top 5 GO terms. The subnetwork depicts the relationships among 5 GO terms and CTD-ILD associated genes. Abbreviations: GO, gene ontology.

The GO and KEGG enrichment analysis of Triptolide intervention CTD-ILD related targets was carried out with R software and the results were visualized. The topological network schematic of target proteins in enriched molecular functions analysed by enrichMap in the clusterProfiler package is listed (Figure 4).

The GO enrichment analysis results obtained a total of 3464 items, The top 20 significantly enriched terms in BP, MF, and CC categories were selected, according to *p* < .05, *p*-values were corrected using the Benjamini–Hochberg procedure. In the biological processes category, the target proteins were mainly involved in Apoptosis, cell oxidative stress, inflammatory response, etc. In the MF category, the target proteins were mainly involved in Receptor ligand binding activity, Ubiquitination of proteins, transcription factor binding, Cytokine activity. In the CC category, the target proteins were classified into the plasma membrane and cell surface, endoplasmic reticulum lumen, etc.

**Figure 5.**
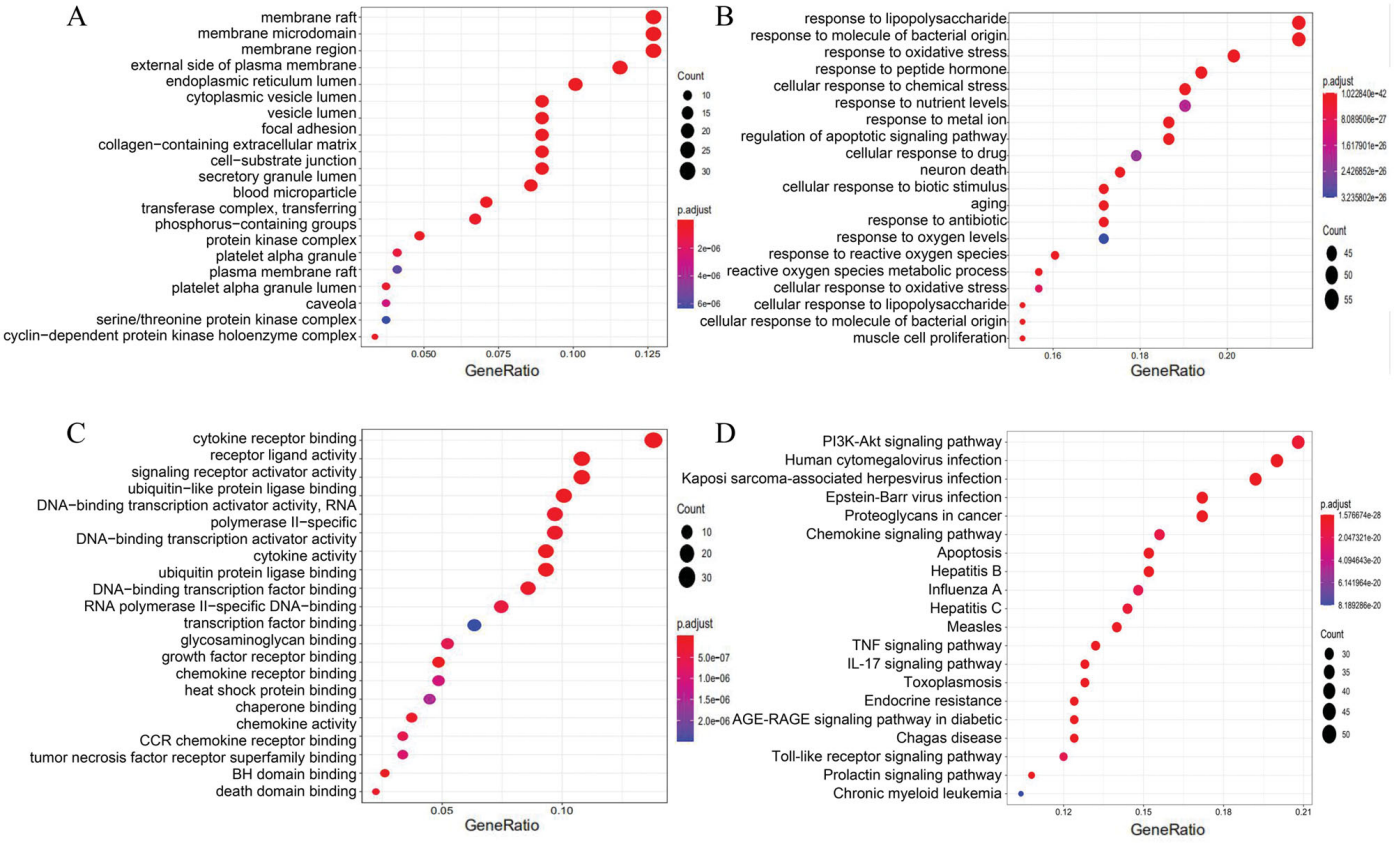
GO and KEGG analysis of genes encoding proteins targeted by Triptolide. GO enrichment analysis identified genes involved in (A) GO-CC analysis, (B) GO-BP analysis, (C) GO-MF Analysis, (D) KEGG analysis. (B) KEGG pathway analyses from bioinformatics data for the molecular signal pathway of Triptolide against CTD-ILD.

There were 173 KEGG enrichment items in total, and the top 20 items were screened according to the KEGG analysis with BH-corrected *p*-values < .05, mainly included PI3K-Akt signalling pathway, multiple virus infections, chemokine signalling pathway, and apoptosis signalling pathway et al. The first 20 related enrichment results were visualized based on GO and KEGG enrichment results (Figure 5).

### 3.6. Molecular docking

**Figure 6.**
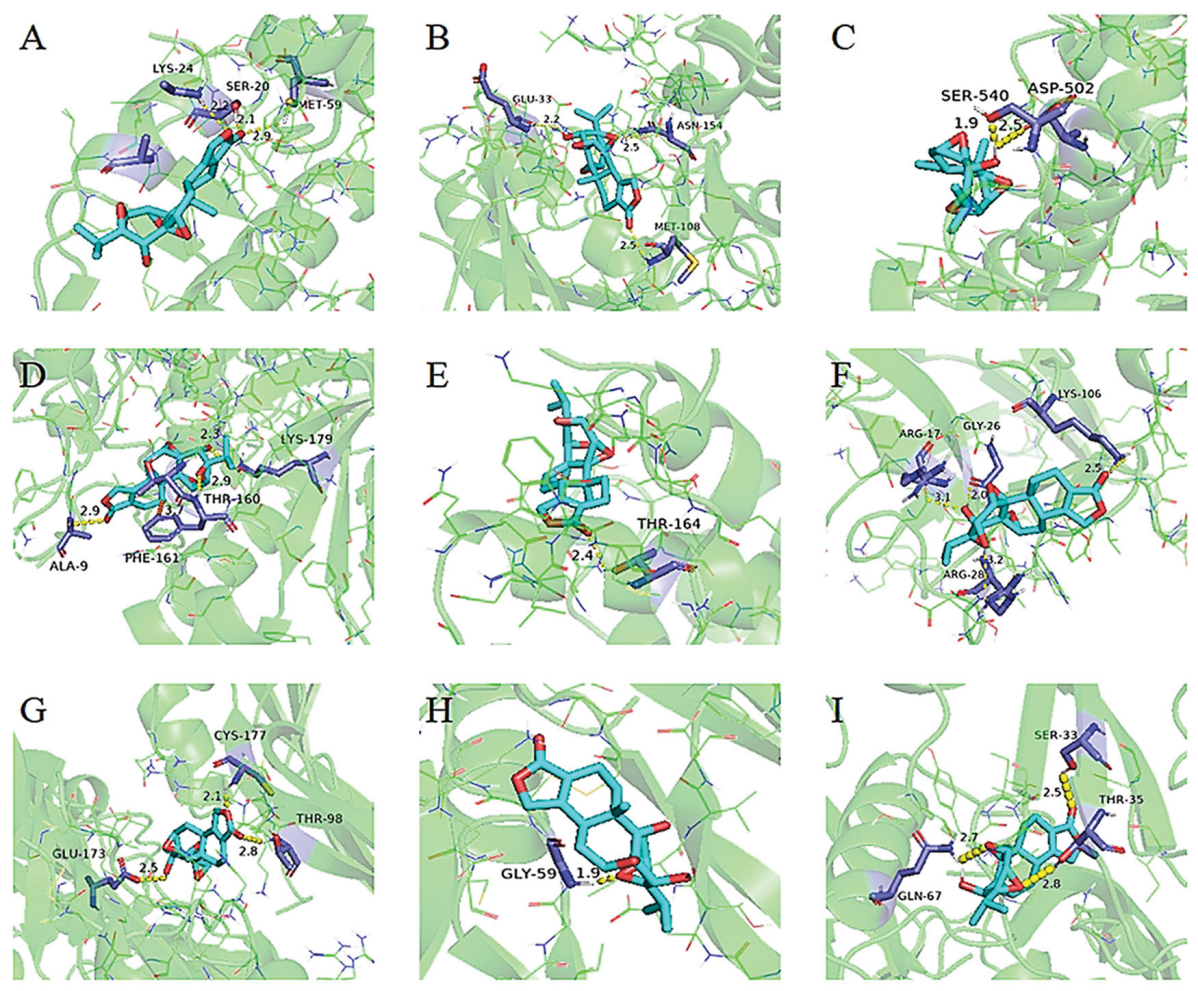
Molecular models of Triptolide binding to its predicted protein targets. Proteins (A)TP53(3DAB), (B) MAPK1(3W55), (C)STAT3(6NUQ), (D)AKT1(6NPZ), (E)IL6(2IL6), (F)FGF(2K8R), (G)PDGF(3MJK), (H) VEGF(1VPF), (I)TGF(3KFD) are shown interacting with a Triptolide molecule, represented by a blue stick model. Lines represent residues in the binding sites. The light dashed lines represent hydrogen bonds, the dark dashed lines demarcate π-π interactions, and the interaction distances are indicated next to the bonds (For interpretation of the references to colour in this figure legend, the reader is referred to the web version of this article).

Molecular docking was conducted between Triptolide, the core components of Tripterygium wilfordii and the key targets. Then the docking results showed that Triptolide could be bound into the docking pocket, with good docking activity between the target proteins. As we tested the following potential target proteins (Figure 6): TP53(PDB: 3DAB), MAPK1(PDB: 3W55), STAT3 (PDB: 6NUQ), AKT1(PDB: 6NPZ), and IL6(PDB: 2IL6), which were high-degree nodes in the interaction network, suggesting they play a critical role in the response to Triptolide in CTD-ILD. Triptolide bound to TP53 by forming hydrogen bonds with SER-20 (length:2.1 Å), MET-59(2.9 Å), and LYS-24 (2.2 Å, Figure 6(A)). There are also 3 hydrogen bonds, GLU-33(2.2 Å), MET108(2.5 Å), and ASN-154(2.5 Å) were predicted between Triptolide and MAPK1(Figure 6(B)). Similarly, Triptolide was predicted to dock into the binding pocket of STAT3 *via* hydrogen bonds SER-540(1.9 Å), ASP-502(2.5 Å, Figure 6(C)). Triptolide was also predicted to dock in the binding pocket of AKT1 *via* multiple hydrogen bonds with THR-160(2.6 Å), LYS-179(2.3 Å), ALA-9(2.9 Å), and π–π interactions with PHE-161 (3.7 Å, Figure 6(D)). Lastly, Triptolide was predicted to dock into the pocket of IL-6 *via* a single hydrogen bond with THR-164 at a distance of 2.4 Å (Figure 6(C)). Furthermore, known protein targets of ILD (FGF, PDGF, VEGF, TGF) was also conducted molecule docking with Triptolide, of whom binding energies were all less than −5kcal·mol^−1^. It is suggested that Triptolide may affect its function by competitively inhibiting the binding of the docking pocket to the target receptor and play an important role in the treatment of CTD-ILD.

## 4. Discussion

Network pharmacology, as a cutting-edge approach provides a full or partial understanding of the principles of network theory and systems biology [27]. This approach has been used to study the pathway “compound-proteins/genes-disease” in a way that captures the complexities among biological systems, drugs, and diseases from a network perspective. Therefore, the network pharmacology research method is used to predict the interrelationship network between drugs and diseases in the study of different fields such as discovering new drugs [28], elaborating pharmacological mechanisms [29], and exploring new targets [30]. In our study, topological analysis of the drug-disease network was conducted, and corresponding molecular docking studies were conducted to enhance the reliability of target prediction conclusions.

**Figure 7.**
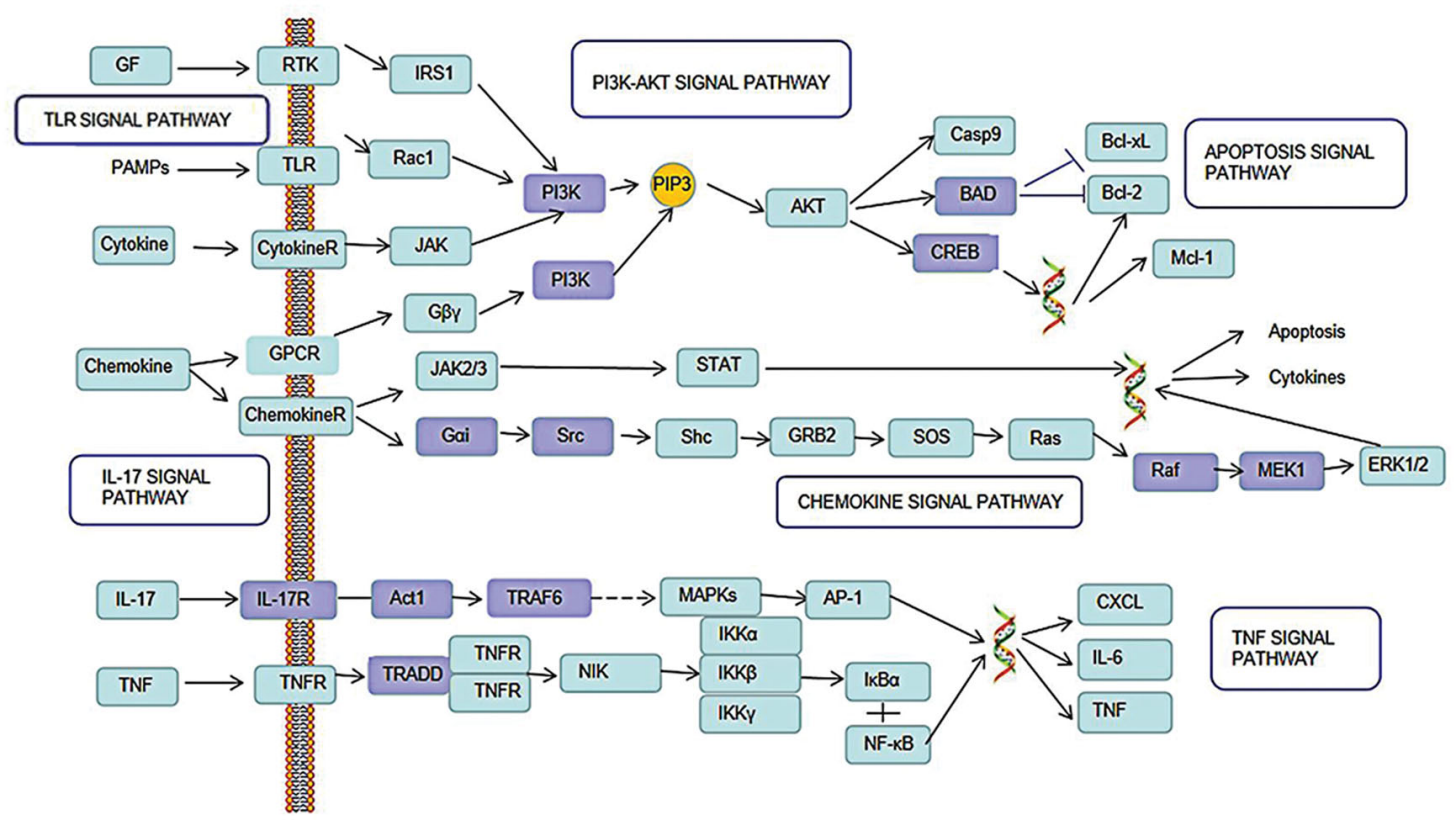
Distribution of the target proteins of Triptolide on the predicted pathway. The dark nodes are potential target proteins of Triptolide, while the light nodes are relevant targets in the pathway.

In the present report, we predict interactions between Triptolide and its potential protein targets by integrating information from publicly available databases about CTD-ILD, as well as elucidating the numerous signalling pathways and networks in which Triptolide targets participate. We also performed docking studies to predict specific interactions between Triptolide and its predicted protein targets. The molecular docking results of Triptolide showed that hydrogen bonding and π-π stacking were the main forms of interaction. Pathway analysis suggested that Triptolide regulates the activation of mainly included PI3K-Akt signalling pathway, multiple virus infections, chemokine signalling pathway, apoptosis signalling pathway, TNF signalling pathway, and IL-17 signalling pathway in CTD-ILD (Figure 7). The results proved that Triptolide can interfere with the process of CTD-ILD, through multiple targets, multiple access. Among them, the top 10 targets are STAT3, AKT1, MAPK1, IL6, TP53, MAPK3, RELA, TNF, JUN, JAK2. By analysis of the hub genes and main significant KEGG pathways, the potential mechanisms of Triptolide in the treatment of CTD-ILD might be attributed to the following aspects.

First of all, These potential targets are closely associated with the progression of pulmonary fibrosis. For example, STAT3 controls crosstalk between epithelial cells and fibroblasts which in turn contributes to disease progression [31]. In fibrogenesis, IL-6 can promote the phosphorylation of STAT3 and its translocation to the nucleus for further gene transcription [32]. JAK2 and STAT3 were verified as a hub pathway to modulating oxidative stress, inflammation, and cell apoptosis in a model of bleomycin-induced acute lung injury rat [33]. c-Jun-N-terminal kinase 1 (JNK1) [34] signalling pathways play an essential role in various physiological and pathological processes such as cell cycle, reproduction, apoptosis, and cellular stress. Recently, it has been found that the transcription factor JUN is highly expressed in pulmonary fibrosis, and its overexpression in mice can induce pulmonary fibrosis. During BLM injury, the pro-inflammatory cytokine such as IL-17A will be up-regulated and mediate the inflammation in the alveolar epithelial cell and also brings about recruitment of certain inflammatory cells in the alveolar surface. IL-17A-mediated p53-fibrinolytic aspects are involved in the regulation of pathogenic progression of acute lung injury (ALI) and pulmonary fibrosis [35].

Second, these targets also participate in the process of epithelial-mesenchymal transition (EMT). EMT is a process in which epithelial cells gradually transform into mesenchymal like cells, losing epithelial function and characteristics. EMT is thought to be involved in the pathogenesis of many lung diseases, ranging from developmental disorders, fibrous tissue remodelling to lung cancer [36]. MAPK3(ERK1), MAPK1(ERK2) belong to ERK signal pathway. *In vitro* and *in vivo* studies have shown that activation of ERK1/2 signalling pathway is involved in TGF-β1-induced EMT [37]. Besides, it’s also necessary for TGF-β1-induced tight junction dissociation and cell migration. Then the expression of downstream fibrosis-related genes were regulated [38]. RELA is one of the major members of the NF-κB family, involved in the transcription and regulation of multiple inflammatory factors. One study confirmed that NF-κB/RelA signalling network plays an important role in type II epithelial mesenchymal transformation in primary airway epithelial cells [39].

Then, the PI3K-Akt signalling pathway, TNF signalling pathway, and IL-17 signalling pathway are known as inflammation-related signalling pathways. In addition, By activating the PI3K-Akt and the mammalian target of rapamycin (mTOR) signalling pathway can inhibit autophagy of bronchial epithelial cells and exacerbate lung damage and fibrosis [40]. Accordingly, inhibiting this pathway can attenuate mitochondrial-dependent apoptosis, endoplasmic reticulum stress, and inflammation in acute lung injury [41]. TNF signalling pathway plays an important role in a variety of connective tissue diseases. Tumour necrosis factor receptors and their corresponding cytokine ligands are involved in many aspects of immune function [42]. Activation of the TNF signalling pathway has been found to induce interstitial lung disease [43]. Interleukin (IL)-17 signal pathway has demonstrated pro-inflammatory effects in chronic inflammation and autoimmune diseases and showed pathogenicity in pulmonary fibrosis are potential therapeutic targets for the treatment of fibroproliferative lung diseases [44]. Interestingly, Signalling pathways are not isolated from each other, such as the IL-17 and Akt pathways have a mutual promoting effect. One study confirmed that IL-17A can inhibit autophagy in keratinocytes by activating the PI3K/Akt/mTOR signalling pathway [45]. Multiple studies have found that viral infection can induce the formation of hyaluronic acid [46] and contribute to a variety of inflammatory cytokines release [47], associated with acute lung interstitial injury [48]. All of them are a costimulatory factors in the progression of pulmonary interstitial disease [49]. Apoptosis was thought to be the only regulated cell death mechanism in the past years [50]. Regulation of cell death is the main mechanism for eliminating damaged, infected, or excess cells, which is also found to be related to the occurrence of ILD [51].

Experimental studies have found that Triptolide has shown good anti-inflammatory and immunosuppressive effects in the treatment of many diseases and verified that Triptolide can attenuate inflammatory response, lung injury, and kidney damage in multiple autoimmune diseases by inhibiting NF-κB, vascular cell adhesion molecule-1, IL-1, IL-6, IL-17, and TNF-α, through interfering NF-κB [10,52], PI3K-AKT-mTOR, and apoptosis signal pathway et al. [53]. So we think that Triptolide may play a vital role in the treatment of CTD-ILD through the above pathway.

Given the uncertainty of the benefits in the present treatment of CTD-ILD, it remains an urgent need for new treatment strategies. we provide several potential targets for CTD-ILD treatment, which could contribute to the development of new therapeutic strategies. Although the therapeutic effect of tripterysium glycosides in the treatment of CTD-ILD has been satisfactory during the past several years. But its mechanism has been rarely studied. This paper is the first study based on the network pharmacology, and molecular docking technology discussed the molecular mechanism of Triptolide, an element of tripterysium glycosides, intervene CTD-ILD. Further vitro and vivo studies are required to explore the mechanism of tripterygium wilfordii and its bioactive constituents in the treatment of CTD-ILD. But, the current research on the treatment of CTD-ILD by Tripterygium wilfordii and its bioactive ingredients is still in the preliminary stage. So, up to date, it’s still a lack of research models to simulate the pathology of CTD-ILD. Last but not least, It is worth noting that tripterygium wilfordii and its active ingredients are toxic to the liver, kidney, or reproductive system. So, how to reduce its toxicity and increase the curative effects should be also taken into consideration.

## Conclusion

The present study analysed the mechanisms underlying the therapeutic effect of Triptolide in CTD-ILD using network pharmacology. Our findings revealed that Triptolide exerts pharmacological effects in CTD-ILD in a multicomponent–multitarget–multipathway manner, including Apoptosis, cell oxidative stress, inflammatory response, and so on. Our findings offer a reference for further investigation of the mechanism underlying the therapeutic effect of Triptolide in CTD-ILD.

## Data Availability

The original contributions presented in the study are included in the article/Supplementary Material, further inquiries can be directed to the corresponding authors.
